# Congenital Midline Cervical Cleft: Diagnosis, Pathologic Findings, and Early Stage Treatment

**DOI:** 10.1155/2012/951040

**Published:** 2012-10-09

**Authors:** Xenophon Sinopidis, Helen P. Kourea, Antonios Panagidis, Vasileios Alexopoulos, Sotirios Tzifas, Gabriel Dimitriou, George Georgiou

**Affiliations:** ^1^Department of Surgery, Karamandanion Children's Hospital, 26331 Patras, Greece; ^2^Department of Pediatric Surgery, University of Patras, 26504 Patras, Greece; ^3^Department of Pathology, University General Hospital of Patras, 26504 Patras, Greece; ^4^Neonatal Intensive Care Unit, University General Hospital of Patras, 26504 Patras, Greece

## Abstract

Congenital midline cervical cleft is a very uncommon malformation of the anterior neck, with less than 100 cases reported in medical literature. Herein we present a case of a female neonate with this anomaly. A detailed description of the macroscopic and microscopic characteristics is performed. As it is derived from the natural history of the lesion, prompt clinical diagnosis, and operative treatment during early infancy predispose to a better aesthetic and functional prognosis.

## 1. Introduction

Congenital midline cervical cleft is an uncommon malformation of the anterior neck with less than 100 cases reported [1–4]. It represents a variant of the cleft category number 30 of the Tessier classification system of craniofacial defects [5]. Though it is always present at birth, it is often overlooked or misdiagnosed. Inadequate treatment may cause secondary complications such as impaired neck extension, microgenia, exostosis, torticollis, or infection [1, 4–8]. Prompt diagnosis and treatment during early infancy leads to a better functional and aesthetic outcome with the least complicated surgical intervention. 

## 2. Case Presentation 

 A female neonate presented with a midline cervical lesion after birth. She was born at the 37th gestation week by caesarian section due to breech presentation and maternal uterine leiomyoma, and weighted 3.010 g. Her parents were healthy and unrelated. There were no karyotypic anomalies. Cervical and spine ultrasound were normal. 

**Figure 1:**
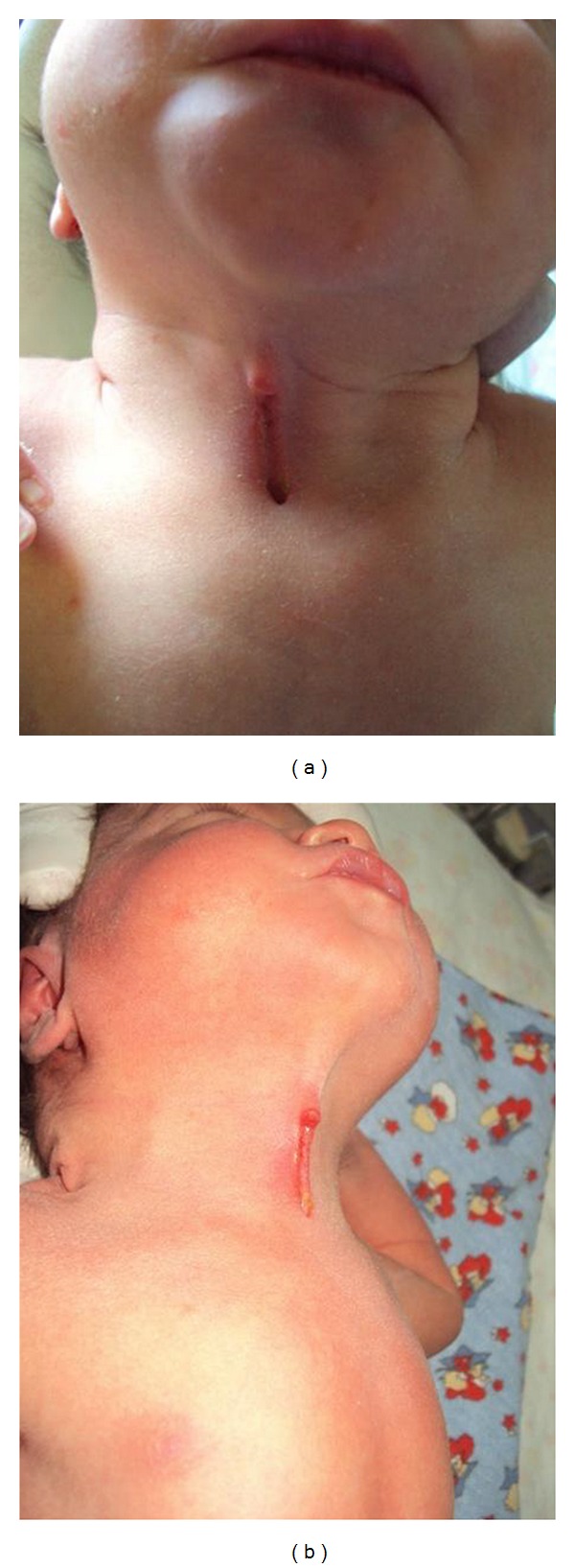
Macroscopic appearance of congenital midline cervical cleft after birth: composed clearly of a superior skin tag, an inferior short caudal sinus and a mucosal sulcus in between (a). Neck extension produces webbing of the skin between the cleft and the mandible (b).

The cervical lesion had the configuration of a linear cleft with a cephalocaudal orientation, extending from the level below the hyoid bone to the suprasternal notch with a length of 2 cm and width of 0.5 cm (Figure 1). It was composed of three components, a notch structure superiorly, a blind sinus of 0.3 cm depth at the inferior end, and a midline longitudinal sulcus between them. There was a seromucinous transparent discharge from its moist pink colored surface which was gradually reduced during the first weeks of infancy. On extension of the neck, a skin web was formed between the cleft and the mandible (Figure 1).

 Surgical excision of the cleft was performed at the age of one month. A longitudinal elliptical incision was performed at the edges of the lesion on healthy skin margins. Special care was given to the complete excision of the caudal sinus and the underlying cephalocaudal fibers which lied under the cleft in the subcutaneous tissue. Continuous absorbable suture was used on a primary closure, without using any of the z-plasty techniques that are reported by other authors [1, 3, 9, 10]. 

**Figure 2:**
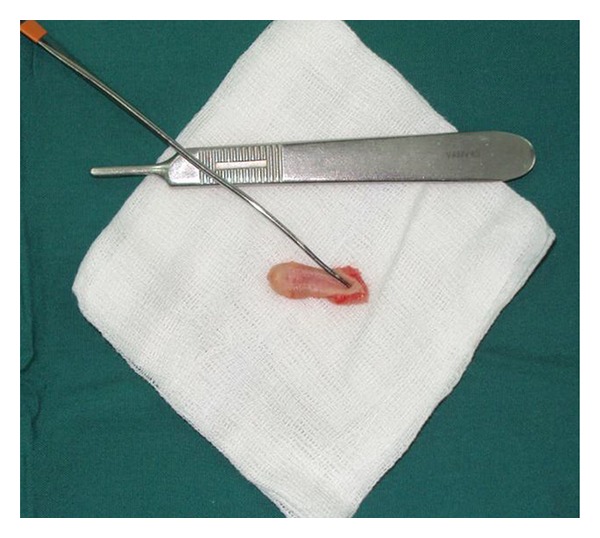
Macroscopic appearance of the excised specimen. The probe is inserted in the caudal blind sinus.

 The excised specimen consisted of a 2.3 × 0.9 × 0.7 cm skin ellipse, the long axis corresponding to the cephalocaudal orientation (Figure 2). The skin papule on the cephalic margin of the malformation measured 0.4 cm. A blind 0.4 cm deep duct was observed at the caudal end of the lesion. The skin surface between these two findings presented a slight linear indentation corresponding to the pathologic tissue of the cleft, measuring 1.6 cm in length. 

**Figure 3:**
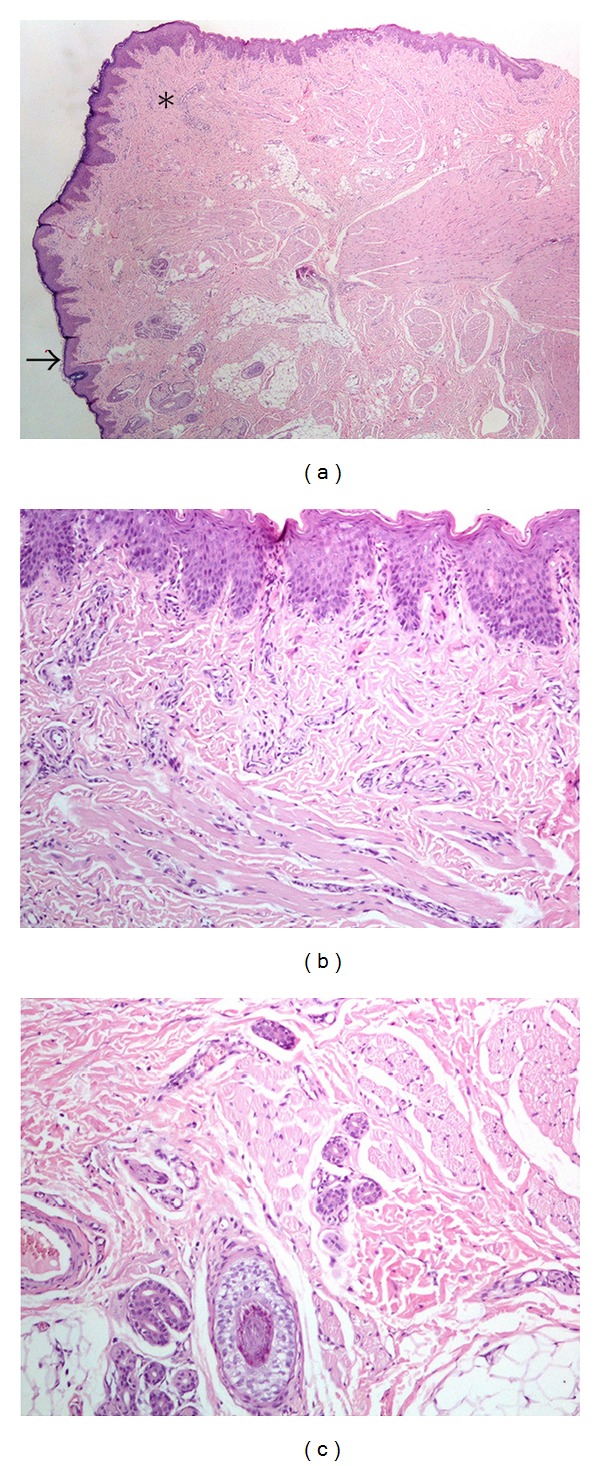
(a) The cephalic papule represented wide bundles of striated muscle covered by stratified squamous epithelium with surface parakeratosis. The arrow shows the transition towards normal skin displaying adnexal structures at the cephalic end of the specimen. The sign “∗” marks the area shown in higher magnification in (b) (×2, hematoxylin-eosin stain). (b) shows thin bundles of striated muscle extending superficially underneath the parakeratotic squamous epithelium covering the papule (×10, hematoxylin-eosin stain). (c) shows the striated muscle bundles at the edge of the lesion to extend towards the adjacent normal skin and to surround sweat gland ducts and a hair follicle (×10, hematoxylin-eosin stain).

 The cephalic papule represented a striated muscle overgrowth covered by stratified squamous epithelium with surface parakeratosis (Figures 3(a) and 3(b)). The striated muscle bundles surrounded sweat gland ducts (Figure 3(c)).

**Figure 4:**
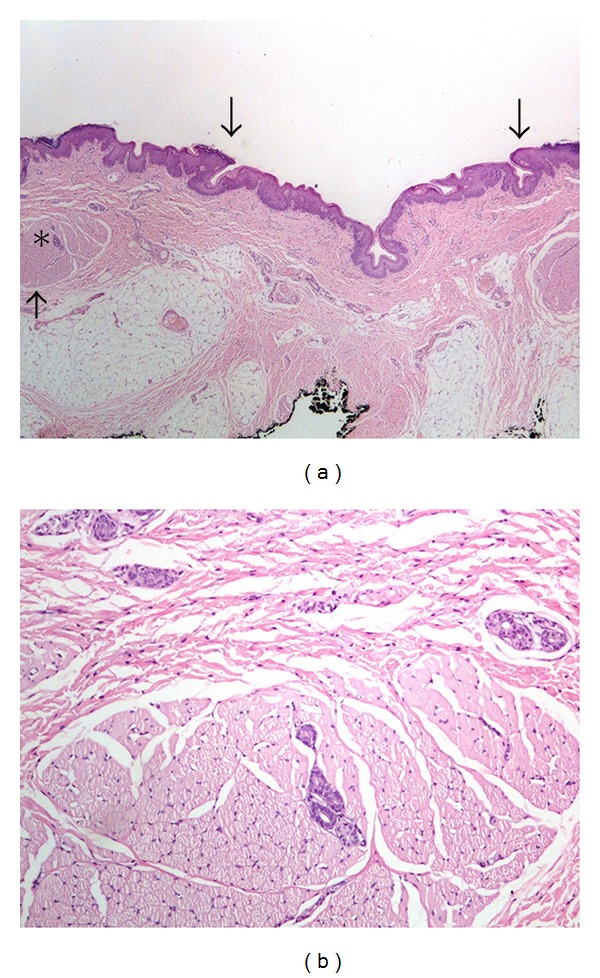
(a) On low power view (×2) the indentation of the skin was also covered by stratified squamous epithelium with surface parakeratosis. The upper arrows point to the transition to normal orthokeratotic epidermis. The underlying dermis showed absence of adnexal structures. The edges of the indentation were marked by the presence of bundles of striated muscle fibers (lower arrow). The “∗” sign marks the area shown in higher magnification in (b) (hematoxylin-eosin stain). (b) Striated muscle bundles at the edges of the skin indentation surround and entrap sweat gland ducts (×10, hematoxylin-eosin stain).

 The linear macroscopic indentation of the epidermis corresponding to the main body of the malformation was covered by stratified squamous epithelium with surface parakeratosis, and was devoid of adnexal structures. The edges of the indentation were marked by the presence of bundles of striated muscle, surrounding the adjacent outer normal skin adnexae (sweat ducts and hair follicles). Another prominent characteristic was the presence of striated muscle in the dermis (Figures 4(a) and 4(b)).

**Figure 5:**
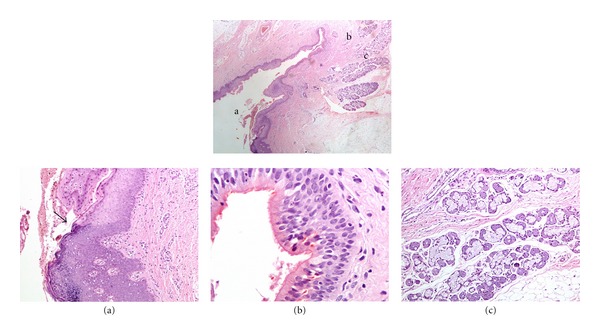
(Top panel) Low power view of the caudal duct (×2). The letters a, b and c represent the foci shown in magnification (a), (b), and (c), respectively (hematoxylin-eosin stain). (a) shows the transition (arrow) between normal epidermis displaying granular layer and orthokeratosis to the parakeratotic squamous epithelium lining the duct (×10, hematoxylin-eosin stain). (b) The lower portion of the blind duct is lined by pseudostratified ciliated epithelium, upper respiratory type (×40, hematoxylin-eosin stain). (c) The duct is surrounded by seromucinous glands (×10, hematoxylin-eosin stain).

The surface portion of the duct at the caudal end of the cleft was lined by stratified squamous epithelium with surface parakeratosis (Figures 5 (top panel) and 5(a)). The lower portion of the duct was lined by pseudostratified ciliated epithelium of upper respiratory type (Figures 5 (top panel) and 5(b)) and was surrounded by seromucinous glands (Figures 5 (top panel) and 5(c)). 

 The pathologic characteristics of the lesion include skin with parakeratotic epidermis, the presence of striated muscle in the dermis, the absence of skin adnexal structures, and the presence of respiratory type epithelium and seromucinous glands (Table 1).

 After a follow-up period of 8 months the patient has a linear cosmetic scar without contracture of the adjacent skin of the neck. During this period the parents were asked to perform stretching exercise manipulation of the neck and apply a scar healing cream of hyaluronic acid. 

## 3. Discussion

 Congenital midline cervical cleft, medial cleft, median fissure of the neck, congenital midline cervical cord, midline cervical webbing, and pterygium colli medianum are many different names given to a malformation that has been reported only 50 times in the English medical literature [1, 3, 10]. Apart from the terminology, the malformation presents interesting points, such as embryologic origin and variety of clinical presentation [9]. A female to male ratio of 2 : 1 is reported, with a sporadic presentation [1, 9, 11]. The lesion is located in the midline of the anterior neck at any point between the mandible and the sternum [1].

 There is a spectrum of clinical presentations [11], however, on its typical presentation, congenital midline cervical cleft consists of three anatomic parts: an superior nipple-like skin tag which hoods a linear area of a red or pink moist surface of atrophic epidermis without adnexal structures, to end to a posterior duct, usually shallow and blind but occasionally going all the way down to the area of the manubrium or the sternum, or towards the hyoid bone [10, 11]. Mucous drain may exit from the inferior duct [9, 11]. The seromucinous discharge resolves gradually the first months of infancy [12, 13]. With time the cleft heals and a longitudinal scar is formed, resulting to the formation of web, which causes contracture of the neck, limits neck mobility, particularly extension, or torticollis [9, 14]. Three clinical outcomes emerge from this evolution. The first is neck contracture and functional compromise, the second is secondary anatomical disarrangement, such as formation of micrognathia, or bony spur (exostosis) of the mandible or sternum, and the third is misdiagnosis later in life, when the cleft achieves the form of a midline linear of spotlike scar, rather than the typical presentation after birth [7–9]. Patients with the lesion were sometimes referred to dermatologists by primary care physicians with the possible diagnosis of a thyroglossal duct cyst or an “unusual birthmark” [15].

 The midline cervical cleft may be a solitary deformity, but there are cases where it is combined with thyroglossal duct cyst, ectopic bronchogenic cyst, branchial cyst, midline hemangioma, ectopia cordis, cleft lip, mandible or tongue, cleft sternum, absence of hyoid bone or thyroid cartilage, or congenital heart disease [9, 12, 16]. This eventually results to a fourth clinical issue, failure of diagnosis of any of these disorders [1].

 Different theories have been proposed on the embryological origin of the congenital midline cervical cleft. Most investigators believe that the defect is the result of fusion failure of the first and second branchial arches in the midline [1, 3, 10, 14, 15]. This theory explains the variations which range from a cord without a cleft to absence of the hyoid bone and thyroid cartilage [14]. Mechanisms proposed to be implicated with incomplete branchial fusion are vascular anomalies (ischemia, necrosis, and scarring), persistence of remnants of the thyroglossal duct and sinus cysts, increased pressure on the cervical area from the pericardial roof in early stages of developing embryo, rupture of a pathologic adhesion between the epithelium of the cardiohepatic fold with that of the ventral part of the first branchial arch, and absence of mesenchymal tissue in the cervical midline [1, 3, 9, 10]. 

 Improper interaction between ectoderm, and mesoderm which may explain the absence of skin adnexal structures, faulty differentiation of mesenchymal tissue, amniotic adhesions, vascular anomalies, exteriorization of a thyroglossal duct remnant and increased pressure in cervical area from the pericardial roof have been also proposed as hypotheses for congenital cleft formation [1, 7, 9, 14]. 

 The proper description of the pathology of a typical congenital midline cleft should be the description of three different anatomic areas. The superior skin tag part may present normal skin, or stratified squamous epithelium with parakeratosis. Presence of cartilage or skeletal striated muscle has been reported [8, 15, 17]. In our case bundles of striated muscle have been located immediately underneath a parakeratotic squamous epithelium covering the papule, in the dermis. 

 Stratified squamous epithelium with surface parakeratosis, continues all the way down the main part of the lesion; combined with the absence of adnexal structures in the underlying dermis, is the hallmark of histological presentation of the major part of the malformation [1, 3, 9]. In our case, striated muscle bundles were present at the edges of the longitudinal part, surrounding and entrapping the adjacent sweat gland ducts of the adjacent normal skin.

 The inferior sinus tract consists of pseudostratified ciliated columnar epithelium with seromucinous glands [1, 8, 9]. Respiratory type epithelium and dense collagen have been described [15]. In our case, it was of upper respiratory type. Rudimentary submucosal salivary gland structures have also been reported [1, 13]. 

 Surgical removal of the cleft is the treatment of congenital midline cervical cleft. Surgery is indicated for cosmetic reasons and the prevention of cervical contracture. Many authors suggest as proper for surgery the age before the second year of life, with earlier repair indicated in more severe cases [9]. The most frequently used techniques are variations of z-type plasty, in order to achieve uncompromised neck extension [1, 3, 4, 9, 10, 12, 15]. We believe that the age of surgical intervention should be early infancy, during the first three months of life. Early repair prevents contracture and cosmetic deformities [4, 18]. The deformity is still producing discharge, and a complete scar is not yet formed. The effect on the function of the neck is still minimal, and as modern neonatal surgery and anesthesia are safe, we believe that there is no reason to postpone surgery. Instead, the possibility to achieve a satisfactory cosmetic and functional result with primary closure, rather than z-plasties who produce a suboptimal scar is greater. 

 In conclusion there are topics to point towards; prompt diagnosis is significant and should not be omitted. Secondary to that, surgical treatment should not be postponed but performed in early infancy. In this age, primary closure technique may have increased possibilities of success. 

## Figures and Tables

**Table 1:** Pathologic findings of the parts of the congenital midline cervical cleft.

Congenital midline cervical cleft portion	Epithelial structures	Mesenchymal structures
Cephalic skin tag	Stratified squamous epithelium with parakeratosis	Striated muscle overgrowth

Midline cleft	Stratified squamous epithelium with parakeratosis and no adnexae	Bundles of striated muscle Striated muscle in the dermis

Caudal end duct	Squamous epithelium with parakeratosis superficially Pseudostratified ciliated epithelium of upper respiratory type at the bottom	Seromucinous glands
